# Point-of-care ultrasound improves the diagnosis of heart failure in patients with dyspnea in primary care

**DOI:** 10.3389/fmed.2026.1721066

**Published:** 2026-02-13

**Authors:** Róbert Kiss-Kovács, Blanka Morvai-Illés, Roland Tóth-Szeles, Ildikó Bakó, András Mohos, Ildikó Ambrus, Luna Gargani, Albert Varga, Gergely Ágoston

**Affiliations:** 1 Department of Family Medicine, University of Szeged, Szeged, Hungary; 2 Primary Care Practice, Szatymaz, Hungary; 3 Primary Care Practice, Ruzsa, Hungary; 4 Primary Care Practice, Szeged, Hungary; 5 Primary Care Practice, Tiszasziget, Hungary; 6 Department of Surgical, Medical and Molecular Pathology and Critical Care Medicine, University of Pisa, Pisa, Italy

**Keywords:** diagnostic accuracy, dyspnea, focused cardiac ultrasound, heart failure, lung ultrasound, point-of-care ultrasound, primary care

## Abstract

**Background:**

Heart failure (HF) is one of the most frequent and clinically important causes of dyspnea, and its recognition in primary care is often hindered by limited access to advanced diagnostics. This study aimed to evaluate whether general practitioners (GPs), after brief training, can use point-of-care ultrasound (PoCUS) to improve the diagnostic accuracy of HF in patients with new-onset dyspnea.

**Methods:**

In this prospective validation study, 112 consecutive adult patients with unexplained new-onset dyspnea were initially enrolled across four primary care practices. After clinical evaluation, 102 patients underwent a standardized lung and focused cardiac ultrasound by GPs, using handheld ultrasound machines. B-line quantification and visual assessment of left ventricular ejection fraction (LVEF) were performed. Diagnoses before and after PoCUS were compared to the final diagnosis established by a cardiologist using standard echocardiography. Agreement metrics and diagnostic accuracy measures were calculated.

**Results:**

Post-PoCUS assessments by GPs showed significantly improved diagnostic performance for HF (sensitivity: 86.8%, specificity: 88.2%, positive predictive value: 93.7%, and negative predictive value: 76.5%) compared to pre-PoCUS clinical judgment (sensitivity: 85.3%, specificity: 38.2%, positive predictive value: 73.4%, and negative predictive value: 56.5%). Agreement with the final diagnosis increased substantially (Cohen’s *κ* from 0.254 to 0.723). GP-assessed B-lines and LVEF correlated strongly with the cardiologist’s results.

**Conclusion:**

A brief, focused training enables GPs to use PoCUS effectively for the detection of HF in patients with dyspnea. Integrating lung and focused cardiac ultrasound into routine primary care may substantially improve diagnostic accuracy, optimize patient management, and reduce unnecessary referrals.

## 1 Introduction

Dyspnea, a subjective experience of breathing discomfort, is a prevalent and diagnostically complex symptom faced in primary care, accounting for approximately 0.9–2.6% of all consultations (1). Its multifactorial etiology poses a significant diagnostic challenge for general practitioners (GPs) (2). Heart failure (HF), a major potential cause of dyspnea, is often underrecognized in primary care, with a prevalence of 15.7% reported among patients aged 65 years or older presenting with exertional dyspnea but without a prior HF diagnosis (3). Given the aging population and improved survival of patients with cardiovascular conditions, the prevalence of HF is expected to increase further, underscoring the importance of timely recognition and accurate diagnosis in primary care (4). Clinical history, physical examination, and electrocardiogram (ECG) often lack the sensitivity and specificity needed to distinguish HF from other causes of dyspnea (5). These limitations are compounded by the fact that acute diagnostic tools such as chest X-ray or natriuretic peptide testing are often unavailable in primary care, further reinforcing the value of point-of-care ultrasound (PoCUS) in this setting. This diagnostic uncertainty can delay appropriate treatment, increase healthcare costs, and worsen patient outcomes (6). PoCUS has emerged as an evolving diagnostic tool in clinical decision-making, including for dyspneic patients (7). Lung ultrasound (LUS), validated in emergency and inpatient settings, enables the rapid detection of pulmonary congestion through the semi-quantitative assessment of B-lines (8–10). Focused cardiac ultrasound (FCU), which includes the assessment of left ventricular systolic function, supplemented with LUS, can help differentiate the causes of dyspnea (11). Current literature on PoCUS in primary care is sparse, with the majority of studies confined to emergency or tertiary care. This gap is critical, as early detection of subclinical and symptomatic pulmonary congestion in stable outpatients could prevent hospitalizations and guide therapy (12). This study addresses these shortcomings by evaluating the diagnostic accuracy of GP-performed combined lung and cardiac PoCUS, specifically B-line assessment and left ventricular systolic function evaluation by visual estimation, to identify HF in dyspneic patients in primary care settings.

## 2 Materials and methods

### 2.1 Study population and screening process

This prospective diagnostic validation study was conducted across four community-based primary care practices in Csongrád-Csanád County, Hungary, between March 2023 and May 2025. Consecutive adult patients aged ≥18 years presenting with new-onset dyspnea of ≤4 weeks’ duration were screened for eligibility. The initial primary care assessment included clinical history, physical examination, vital signs, and a resting electrocardiogram. Patients were not enrolled if a clear and clinically sufficient etiological explanation for dyspnea could be confidently established at presentation, including respiratory tract infection, acute exacerbation of COPD or asthma, musculoskeletal or anxiety-related dyspnea, or symptomatic anemia. The 4-week symptom duration threshold was selected to capture newly developed or acutely worsening dyspnea consistent with suspected heart failure. Additional predefined exclusion criteria included active malignancy, neuromuscular disorders affecting respiratory mechanics, and significant thoracic deformities that could impair ultrasound image acquisition or interpretation. Patients with a prior cardiologist-confirmed diagnosis of HF were excluded to ensure the inclusion of newly suspected cases. Patients presenting with critical or unstable clinical status (e.g., hemodynamic instability, severe hypoxemia, rapidly progressive dyspnea, or signs of impending respiratory failure) were also excluded and were urgently referred to tertiary-level emergency care. Patients with multimorbidity, including chronic kidney disease, interstitial lung disease, pulmonary arterial hypertension, or fluid overload states (e.g., liver cirrhosis with ascites, nephrotic syndrome, hypoalbuminemia, or lymphangiectasia), were also included to reflect the clinical complexity of real-world primary care practice.

### 2.2 GP training and PoCUS acquisition protocol

The four participating GPs were board-certified physicians without prior formal ultrasound training. Before patient enrollment, they completed a standardized, competency-based PoCUS training program delivered by cardiologists certified by the European Association of Cardiovascular Imaging (EACVI), in accordance with EACVI consensus statements on lung ultrasound in heart failure and the EACVI core curriculum for FCU (10, 13). Training consisted of video-based learning with 20 annotated lung and cardiac ultrasound cases (covering both normal and abnormal B-line patterns and normal and reduced LVEF), hands-on supervised scanning of 20 consecutive heart failure patients in a tertiary cardiology unit, and a review of image acquisition protocols. Competency was confirmed once GPs consistently demonstrated the ability to obtain diagnostic-quality apical four-chamber (A4Ch) views and to identify and quantify B-lines across eight thoracic zones. All PoCUS examinations were performed using GE Vscan Air CL handheld ultrasound devices (GE Healthcare, Norway), equipped with a convex transducer (2–5 MHz) (10, 14). This device is a Food and Drug Administration (FDA)-cleared and Conformité Européenne (CE)-marked diagnostic tool for PoCUS. Although the probe is convex, the device includes a dedicated cardiac preset optimized for qualitative FCU image acquisition, enabling visual estimation of left ventricular systolic function. LUS was performed in the supine position using a standardized eight-zone protocol (15). B-lines were defined according to international criteria as discrete, laser-like vertical artifacts arising from the pleural line, extending to the screen edge without fading, and moving synchronously with respiration (8). Semi-quantitative scoring followed established practice, with individual B-lines counted (0–10 per zone); when B-lines were confluent, the percentage of the screen occupied by B-lines below the pleural line was divided by 10 (e.g., 70% = 7 B-lines) (10, 16). Pulmonary congestion was defined as the presence of B-lines consistent with extravascular lung water in a minimum of two lung zones, where each zone met the criterion of ≥3 B-lines visualized in a longitudinal intercostal plane between two ribs (8). FCU was performed in the left lateral decubitus position, and the A4Ch view was recorded over 3 s. Left ventricular systolic function was visually estimated as normal (LVEF ≥50%) or reduced (LVEF <50%) according to the European Society of Cardiology (ESC) guidelines, using the eyeballing technique (13, 17).

### 2.3 Data collection by GPs

GPs completed a structured questionnaire for each patient, recording pre- and post-PoCUS diagnoses and key clinical data, including demographics, anthropometrics, comorbidities, medications, symptoms, the number of B-lines across thoracic zones, left ventricular systolic function, and whether diuretics were initiated during the visit. Dyspnea severity was also assessed using the modified Borg scale (0: no breathlessness; 10: maximal breathlessness), a validated tool for standardizing patient-reported symptoms during the initial consultation (18).

### 2.4 Reference standard and blinded cardiologist assessment

Following the GP-performed PoCUS examination, all enrolled patients underwent independent evaluation by a cardiologist who was entirely blinded to GP clinical assessments, PoCUS findings, and related information. Cardiology reassessments were scheduled within approximately 1 week of the initial primary care visit, reflecting the realistic capacity and referral patterns of the cardiology outpatient clinic. Transthoracic echocardiography was performed using a GE Vivid S70N ultrasound system equipped with a phased-array probe (1.5–3.6 MHz). LVEF was quantified using the modified biplane Simpson method as a criteria-forming component of HF diagnosis, and a full standard echocardiographic dataset, including chamber dimensions, wall motion, mitral inflow patterns, tissue Doppler velocities, and right-sided parameters, was recorded in accordance with the 2022 EACVI recommendations (19). B-lines were assessed using the same eight-zone protocol for consistency but were recorded as ancillary findings only and not used as primary diagnostic criteria to avoid incorporation bias. N-terminal pro-B-type natriuretic peptide (NT-proBNP) concentrations were obtained using age-adjusted cutoffs to help differentiate HF from non-cardiac causes of dyspnea. The adjudicated reference diagnosis of HF was established by the blinded cardiologist strictly according to the 2021 European Society of Cardiology (ESC) guidelines, integrating transthoracic echocardiography, NT-proBNP values, and structured clinical evaluation (17). Cardiologist findings, such as exertional dyspnea, orthopnea, pulmonary crackles, or lower-extremity edema, were considered essential components of the diagnostic assessment. When a definitive diagnosis could not be established or non-cardiac pathology was suspected, patients were referred for further specialist evaluation (e.g., internal medicine specialist, pulmonologist, nephrologist, hematologist, or rheumatologist) based on the predominant clinical suspicion derived from medical history, physical examination, and baseline laboratory findings suggestive of pathology within the respective organ system.

### 2.5 Diagnostic evaluation and outcome measures

Before PoCUS, GPs recorded a working diagnosis based on conventional diagnostic methods, including medical history, physical examination, and ECG findings. After integrating the findings from LUS and FCU, a revised diagnosis was recorded to reflect the real-world decision-making process in primary care. Pre- and post-PoCUS diagnoses were compared to evaluate the impact of PoCUS on diagnostic accuracy, with a particular focus on identifying HF. In patients ultimately diagnosed with HF, the number correctly identified by GPs before and after PoCUS was assessed. Visual LVEF classification (normal vs. reduced) by GPs was compared to the cardiologist’s reference. B-line counts were compared between providers, acknowledging the possibility of temporal variability due to diuretic therapy or spontaneous short-term changes. However, provider comparisons were used solely to quantify interobserver agreement and short-term measurement divergence and were not incorporated into the reference standard; temporal variability was assessed separately by stratifying B-line changes according to interim diuretic exposure, ensuring independence of outcome measurement from diagnostic adjudication.

### 2.6 Statistical analysis

All statistical analyses were conducted using IBM^®^ SPSS^®^ Statistics version 29 (SPSS, Chicago, IL, USA). Continuous variables were expressed as means ± standard deviation. Categorical variables were summarized as frequencies and percentages. Between-group comparisons were performed using t-tests or Mann–Whitney *U* tests for continuous variables and chi-squared or Fisher’s exact tests for categorical variables. Diagnostic agreement was evaluated using Cohen’s kappa or intraclass correlation coefficients. Correlation analyses were performed using Spearman’s rank correlation. In addition, sensitivity, specificity, positive predictive value (PPV), and negative predictive value (NPV) were evaluated. A receiver operating characteristic (ROC) curve analysis was conducted with area under the curve (AUC) estimation. A two-sided *p*-value of <0.05 was considered statistically significant.

## 3 Results

### 3.1 Study population and baseline characteristics

**Figure 1:**
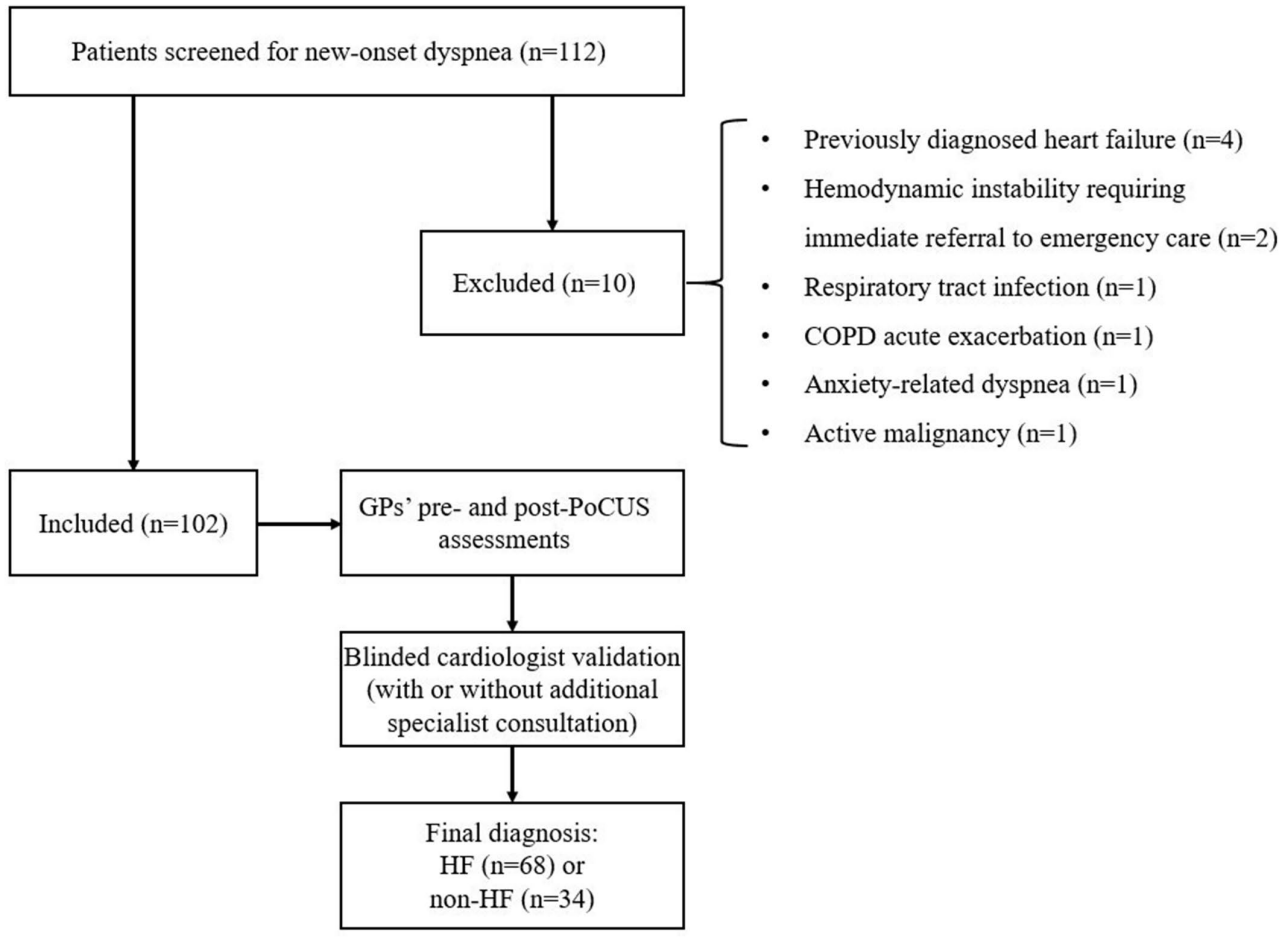
Study flowchart. Flow diagram depicting patient screening, exclusions, inclusion in the final study cohort, and the diagnostic pathway leading to the final adjudicated diagnosis of HF or non-HF. COPD, chronic obstructive pulmonary disease; GP, general practitioner; HF, heart failure; PoCUS, point-of-care ultrasound.

After the application of the predefined inclusion and exclusion criteria, 10 patients who presented with new-onset dyspnea were excluded, resulting in a final study population of 102 participants, as shown in Figure 1. The final cohort consisted of 60 women (58.8%) and 42 men (41.2%) with a mean age of 69.1 ± 13.0 years.

The mean interval between the GPs’ and the cardiologist’s assessments was 3.7 ± 2.3 days. This interval varied due to high patient volume at the cardiology outpatient clinic. The main characteristics of the patients are detailed in Table 1. Patients with HF were older, exhibited a higher prevalence of cardiovascular comorbidities—particularly coronary artery disease and chronic kidney disease—and had substantially higher NT-proBNP levels compared with those without HF. Atrial fibrillation was also more common in the HF group. No other baseline variables differed significantly between the two groups. Further patient characteristics and clinical variables are presented in Supplementary Table S1. An overview of the clinical and echocardiographic parameters in patients with and without cardiologist-confirmed HF is provided in Supplementary Table S2.

**Table 1:** Main characteristics of patients in the overall study population, stratified by the final diagnosis of heart failure.

Patient characteristics and clinical variables	All patients (*n* = 102)	Patients with cardiologist-confirmed HF (*n* = 68)	Patients without cardiologist-confirmed HF (*n* = 34)	*p*-value
Demographics and anthropometrics				
Age, years	69.1 ± 13.0	72.2 ± 9.8	62.9 ± 16.1	NS
Male	42 (41.2%)	32 (76.2%)	10 (23.8%)	NS
Body mass index, kg/m^2^	29.6 ± 5.5	29.4 ± 4.8	30.0 ± 6.6	NS
Key comorbidities				
Hypertension	88 (86.3%)	62 (70.5%)	26 (29.5%)	NS
Diabetes mellitus	34 (33.3%)	27 (79.4%)	7 (20.6%)	NS
Coronary artery disease	32 (31.4%)	26 (81.2%)	6 (18.8%)	0.035
Chronic obstructive pulmonary disease	12 (11.8%)	7 (58.3%)	5 (41.7%)	NS
Chronic kidney disease	27 (26.5%)	24 (88.9%)	3 (11.1%)	0.004
Key laboratory parameters				
NT-proBNP, pg./mL	1702.2 ± 2689.0	2493.6 ± 2992.6	119.4 ± 146.3	<0.001
Creatinine clearance, mL/min/1.73m^2^	66.2 ± 18.9	60.4 ± 18.9	77.8 ± 12.7	<0.001
Key ECG findings				
Sinus rhythm	71 (69.6%)	38 (53.5%)	33 (46.5%)	<0.001
Atrial fibrillation	31 (30.4%)	30 (96.8%)	1 (3.2%)	<0.001
HF phenotypes				
HFpEF, *n* (%)	–	50 (73.5%)	–	–
HFrEF, *n* (%)	–	18 (26.5%)	–	–

Values are present as mean ± standard deviation (SD) or number (%). ECG, electrocardiogram; HF, heart failure; HFpEF, heart failure with preserved ejection fraction; HFrEF, heart failure with reduced ejection fraction; NT-proBNP, N-terminal pro-B-type natriuretic peptide; NS, not significant.

### 3.2 Agreement between GP and cardiologist assessments

The agreement between each GP (GP1–GP4) and the cardiologist in B-line quantification was excellent. The intraclass correlation coefficients (ICCs) (single measures) were as follows: GP1 ICC = 0.904 (95% CI: 0.807–0.952; *p* < 0.001), GP2 ICC = 0.919 (95% CI: 0.826–0.963; *p* < 0.001), GP3 ICC = 0.924 (95% CI: 0.831–0.967; *p* < 0.001), and GP4 ICC = 0.868 (95% CI: 0.701–0.945; *p* < 0.01). Regarding the categorization of LVEF, the agreement between GPs and the cardiologist showed varying levels of consistency. Substantial agreement was observed for GP1 (Cohen’s *κ* = 0.715; 95% CI: 0.413–1.000; *p* < 0.001), GP3 (*κ* = 0.777; 95% CI: 0.360–1.000; *p* < 0.001), and GP4 (*κ* = 0.875; 95% CI: 0.638–1.000; *p* < 0.001). However, GP2 exhibited only fair agreement, with a non-significant Cohen’s *κ* = 0.324 (95% CI: −0.137–0.785; *p* = 0.102).

### 3.3 Impact of PoCUS on diagnostic accuracy

**Figure 2:**
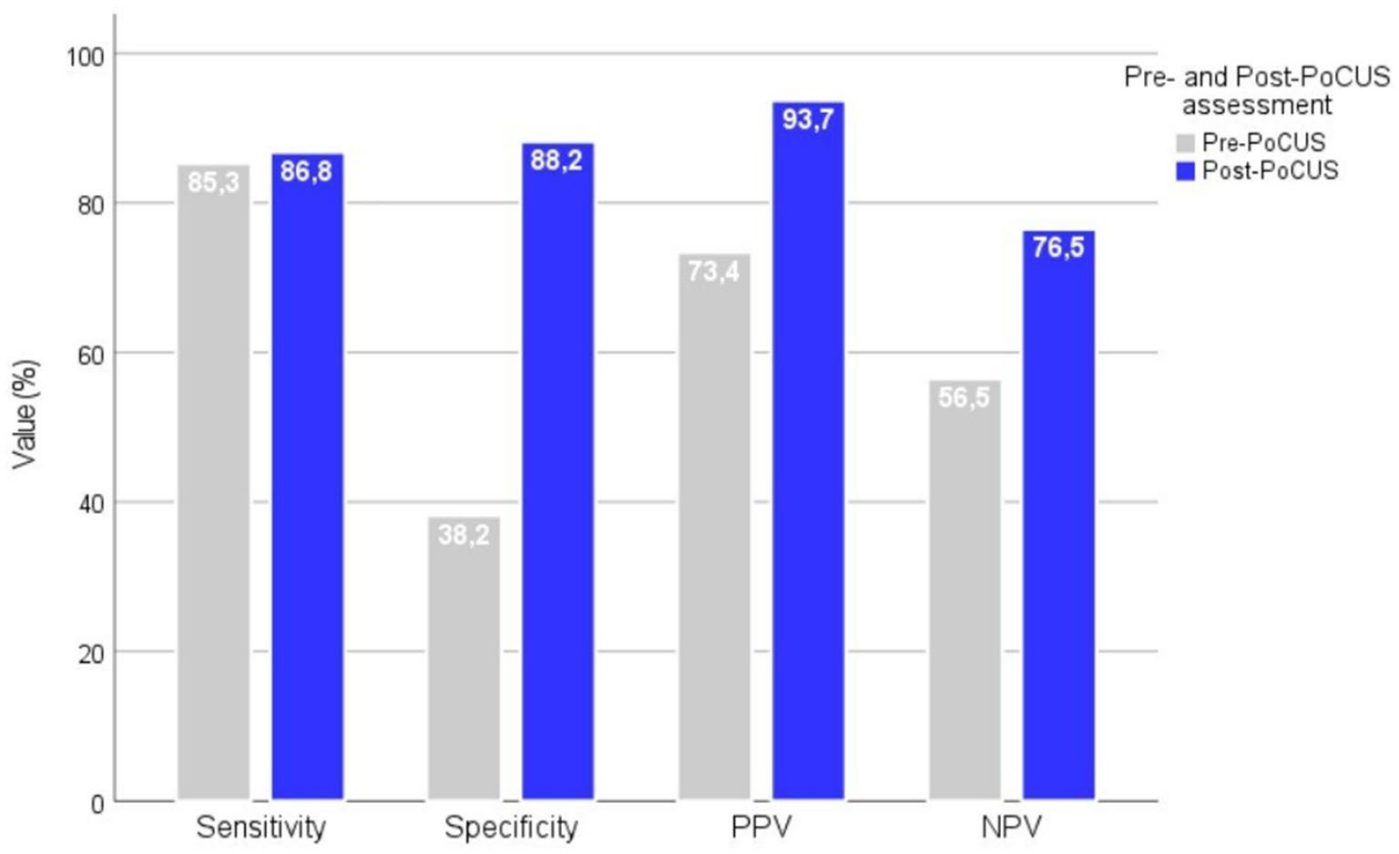
Diagnostic performance metrics before and after PoCUS assessment by GPs. Diagnostic performance metrics (sensitivity, specificity, PPV, and NPV) of GPs before and after the implementation of PoCUS in the diagnostic workflow. The application of PoCUS significantly improved all diagnostic parameters. PPV, positive predictive value; NPV, negative predictive value.

Compared to the final diagnosis, the pre-PoCUS clinical assessment by GPs revealed moderate sensitivity (85.3%) but low specificity (38.2%) and a weak yet statistically significant agreement (Cohen’s *κ* = 0.256; 95% CI: 0.060–0.452; *p* = 0.007). The PPV of the pre-PoCUS assessment was 73.4%, while the NPV was 56.5%. Compared to the final diagnosis, the post-PoCUS clinical assessment by GPs showed markedly improved diagnostic performance, with a sensitivity of 86.8%, a specificity of 88.2%, a PPV of 93.7%, and an NPV of 76.5%. These findings are illustrated in Figure 2. The level of agreement between post-PoCUS diagnosis and the reference diagnosis was substantial (Cohen’s *κ* = 0.723; 95% CI: 0.584–0.862; *p* < 0.001).

**Figure 3:**
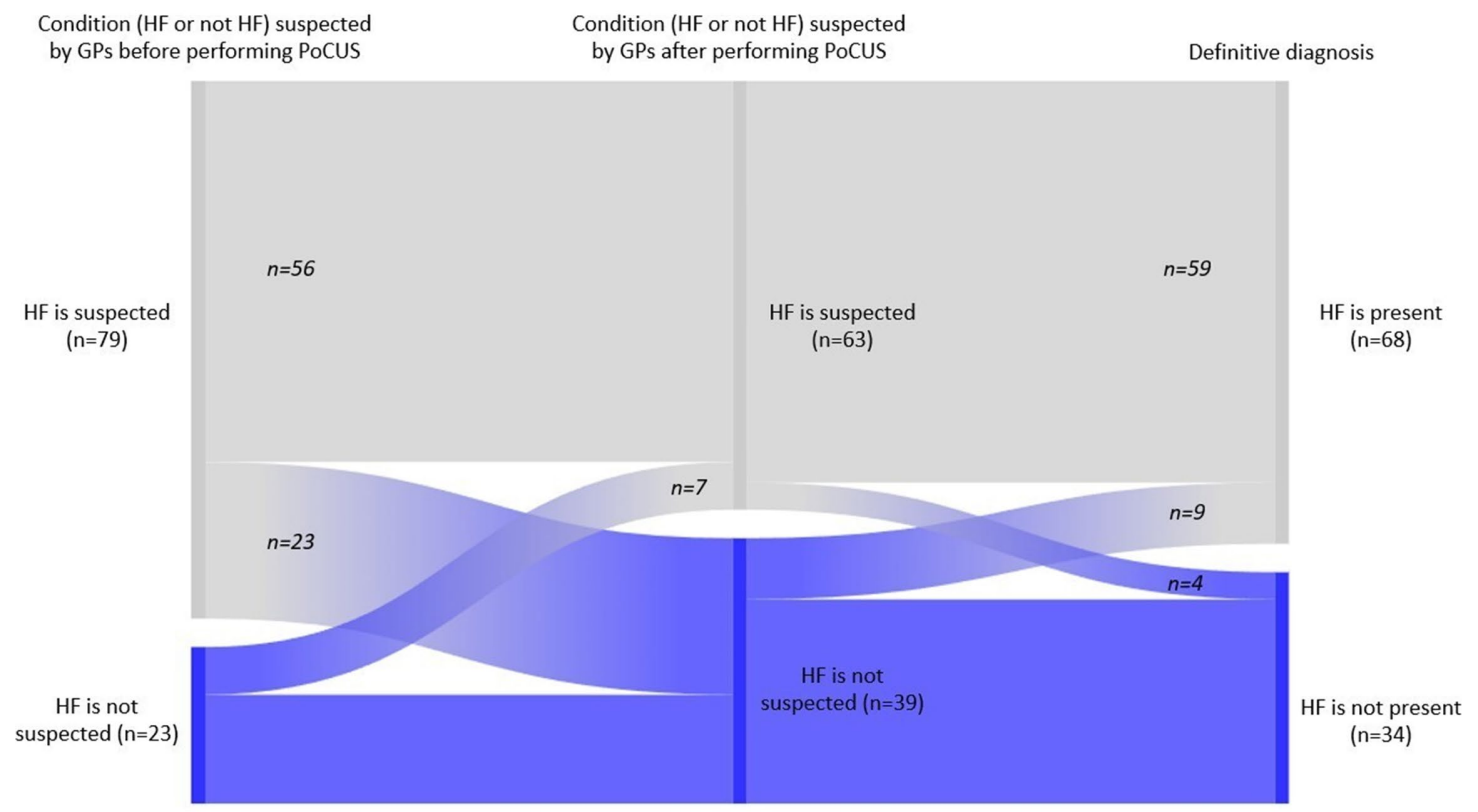
Impact of PoCUS on GPs’ clinical suspicion of heart failure compared to the validated final diagnosis. Alluvial diagram illustrating the reclassification of patients by GPs before and after, compared with the final cardiologist-confirmed diagnosis of HF. The figure highlights reductions in overdiagnosis and improvements in the detection of true HF cases. HF, heart failure.

A multi-step alluvial diagram visually depicts how PoCUS impacted GPs’ clinical suspicion of HF relative to the final diagnosis, as shown in Figure 3. Before PoCUS, GPs suspected HF in 79 patients (77.5%) and ruled it out in 23 patients (22.5%). Post-PoCUS, 23 patients (22.5%) were reclassified from HF to non-HF, and 7 (6.9%) were reclassified from non-HF to HF. According to the final diagnosis, 68 patients (66.7%) had HF, and 34 patients (33.3%) did not have HF. These changes resulted in improved diagnostic accuracy, as the number of HF-suspected cases decreased from 79 to 63 (−20.3%), thereby reducing the incidence of overdiagnosis. In comparison, the detection of previously unrecognized HF cases increased (30.4% of initially non-HF patients were correctly reclassified).

Among patients with a final diagnosis of HF (*n* = 68), the underlying causes were chronic cardiovascular conditions, namely coronary artery disease (*n* = 25; 36.8%), atrial fibrillation (*n* = 18; 26.5%), valvular heart disease (*n* = 9; 13.2%), cardiomyopathy (*n* = 8; 11.8%), hypertensive heart disease (*n* = 6; 8.8%), and pulmonary arterial hypertension (*n* = 2; 2.9%). The ROC curves for the diagnostic performance of NT-proBNP and GP-assessed B-line count in HF are presented in Supplementary Figure S1. Box plots illustrating the distributions of clinical parameters (B-lines assessed by the GP and by the cardiologist, NT-proBNP levels, and E/e’ ratio), stratified according to final HF diagnosis, are presented in Supplementary Figure S2. Non-cardiac causes of dyspnea in patients without a final diagnosis of heart failure summarize the full spectrum of alternative etiologies identified among non-HF patients, as presented in Supplementary Table S3.

### 3.4 Exploratory stratified analysis by pre-referral diuretic treatment

An exploratory stratified analysis was conducted comparing patients who received diuretic therapy (*n* = 28; 27.5%) and those who did not (*n* = 74; 72.5%) prior to cardiologist evaluation, acknowledging the interval between the GPs’ and the cardiologist’s assessments. Since B-line distributions were non-normal, the Mann–Whitney *U* tests were used. Cardiologist-assessed total B-line counts were significantly lower in patients who received diuretics prior to reassessment compared with those who did not receive diuretics (*U* = 468.0; *z* = −4.28; *p* < 0.001), indicating a measurable reduction of pulmonary congestion between evaluations. Moreover, the absolute discrepancy between GP- and cardiologist-derived B-line counts was significantly smaller in the diuretic-treated patients (*U* = 163.5; *z* = −6.62; *p* < 0.001), suggesting that treatment initiation systematically narrowed interobserver differences.

## 4 Discussion

### 4.1 Comparison with previous studies

This study assessed how LUS and FCU, performed by GPs after brief structured training, influenced the diagnostic accuracy of HF in patients with new-onset dyspnea. PoCUS notably improved specificity, sensitivity, and diagnostic agreement with final cardiologist-confirmed outcomes. B-line quantification and visual LVEF estimation showed strong concordance with specialist assessments, supporting the feasibility and clinical value of PoCUS in routine primary care. The findings of this study align closely with the growing body of literature supporting the diagnostic value of PoCUS, particularly LUS and FCU, in assessing HF (20–23). Several studies have found that B-lines identified via LUS are strongly associated with elevated pulmonary capillary wedge pressure and correlate well with pulmonary congestion in patients with acute decompensated HF (24, 25). A meta-analysis reported that the presence of diffuse B-lines on LUS had a pooled sensitivity of 94% and a specificity of 92% for the diagnosis of acute HF in emergency settings, exceeding the diagnostic performance of chest radiography and comparable to natriuretic peptide (26). Our findings mirror these results, as both GP- and cardiologist-assessed B-line counts were significantly higher in patients with HF. Notably, the diagnostic contribution of B-lines remained robust even in a heterogeneous primary care population, in which comorbidities such as chronic lung disease or renal impairment are frequent and can complicate clinical assessment. This is supported by a previous outpatient study, which found that LUS performed by trained GPs provided excellent diagnostic accuracy in distinguishing HF from other causes of dyspnea (20). In addition to LUS, our study showed that visual estimation (“eyeballing”) of LVEF by GPs had high concordance with cardiologist-performed quantitative echocardiography using Simpson’s biplane method. This finding is consistent with prior literature, which shows that brief, structured training enables non-cardiologists to estimate LVEF with substantial agreement (27). Previous studies have shown that GPs could reach good agreement with cardiologists on LVEF estimation after a focused training course, supporting the inclusion of such skills in generalists (23, 28). Our results reinforce the potential of eyeballing LVEF as a practical and accessible tool for the identification of HF with reduced ejection fraction in primary care. The majority of existing studies evaluating integrated LUS and FCU approaches have been conducted in hospital or emergency settings (29, 30). For instance, a study combined a lung and focused cardiac ultrasound protocol, which improved diagnostic confidence and accuracy in differentiating between cardiac and pulmonary causes of dyspnea in emergency department settings (31). Our study extends these findings into primary care, showing that GPs, following short training, can apply a similar combined approach with high accuracy in outpatient settings. Our findings are consistent with previous studies showing that GPs can acquire lung ultrasound and visual LVEF estimation skills through brief, focused training (32, 33). The potential for LUS to support not only diagnosis but also therapeutic monitoring and clinical follow-up is another area of emerging interest (34, 35). Prior studies have shown that B-line burden responds dynamically to diuretic therapy, often decreasing within hours after effective decongestion. Changes in B-line number predicted readmission in patients discharged after HF hospitalization (12, 36). Although our study did not primarily focus on longitudinal monitoring, we documented the timing of diuretic administration relative to specialist reassessment. We observed a consistent reduction in B-line counts at specialist reassessment among patients who received diuretics during the pre-referral interval, suggesting sonographic responsiveness to treatment. These findings confirm that both the referral interval and the initiation of diuretics can meaningfully influence sonographic markers of pulmonary congestion quantified via B-line metrics, although these temporal effects did not materially alter the principal conclusions of the study. This supports the idea that PoCUS, particularly LUS, can be used not only for diagnosis but also for monitoring the effectiveness of diuretic therapy in primary care settings. Overall, our results align with previous literature regarding the diagnostic performance of PoCUS but contribute novel data by demonstrating its feasibility and clinical value specifically in primary care (37, 38). The pragmatic design and real-world implementation strengthen the relevance of our findings, which suggest that GPs can incorporate PoCUS into their diagnostic workflow to improve the evaluation of patients with new-onset dyspnea and suspected HF. Taken together, these findings reinforce the growing consensus that PoCUS is both a feasible and valuable diagnostic tool in the hands of trained GPs.

### 4.2 Implications for research, clinical practice, education, and policy

These findings suggest that PoCUS can serve as a valuable diagnostic adjunct in primary care by aiding in the identification or ruling out HF in patients with dyspnea. It empowers GPs to make more informed decisions at the point of care, potentially reducing diagnostic delays and unnecessary referrals. In patients with pre-existing lung disease, such as COPD, the interpretation of B-lines requires particular clinical context. While COPD is typically characterized by a “dry lung” ultrasound pattern, the absence of B-lines may be clinically informative by supporting the exclusion of pulmonary congestion, even when symptoms such as dyspnea are not clear. In our cohort, dry lung findings on PoCUS often contributed to increased diagnostic confidence when ruling out heart failure in patients with known COPD. However, this observation is based on clinical interpretation rather than the formal subgroup analysis. PoCUS findings should therefore always be interpreted in conjunction with clinical assessment and complementary investigations. The observed 20.3% reduction in HF-suspected cases after PoCUS implementation may have important downstream implications by reducing unnecessary rule-out echocardiography and specialist referrals, which are recognized cost drivers in primary care. The feasibility of training GPs to competence within a short timeframe also opens opportunities for integrating PoCUS education into primary care residency programs and continuing medical education. Although the GPs participating in this study received their initial hands-on training within a tertiary cardiology center, this reflects the study setting rather than a structural limitation of the training model. Recently, a government-supported, competency-based PoCUS training program has been launched in Hungary with the explicit aim of extending access to structured ultrasound education to GPs practicing outside major academic centers. Within this program, GPs working in geographically remote or rural practices are required to complete a 2-day intensive, in-person practical training course at designated university-based training sites. Following this initial hands-on phase, participants continue structured case collection within their own practices, during which telemedicine-based consultation with supervising radiologists and cardiologists is available to support image acquisition, interpretation, and quality assurance. This blended educational framework combines centralized, short-duration practical training with decentralized, practice-based skill development, thereby reducing long-term reliance on continuous hospital-based supervision and supporting the scalability of PoCUS training in primary care. Nevertheless, in resource-limited settings where access to ultrasound devices or teleconsultation infrastructure is constrained, the scalability of such programs may remain limited. From a policy perspective, our results support the inclusion of handheld ultrasound devices in primary care reimbursement frameworks and national diagnostic strategies, especially in settings with limited access to specialist imaging. Future research should evaluate the cost-effectiveness, long-term clinical outcomes, and patient perspectives associated with the use of PoCUS in primary care.

### 4.3 Strengths and limitations

#### 4.3.1 Strengths

A major strength of this study is its pragmatic real-world design, conducted in routine primary care practices where diagnostic uncertainty is common, and multimorbidity frequently complicates decision-making. The inclusion of patients presenting with new-onset dyspnea reflects the true spectrum of presentations encountered in primary care. This study also shows the feasibility of integrating FCU and LUS into frontline primary care, using equipment and operator training conditions that closely mirror everyday GP practice. Importantly, the findings provide clinically relevant insights into how PoCUS may modify diagnostic reasoning in a real-world setting, supporting its potential to improve diagnostic accuracy in patients with suspected HF.

#### 4.3.2 Limitations

This study has some limitations. First, it was conducted within a single geographic region, which may restrict the generalizability of the findings. The sample size, although adequate for the primary analysis, did not allow for more detailed subgroup evaluations. Moreover, the enrollment of this cohort over 2 years across four practices may reflect a selection process in which individuals with clearly identifiable conditions or urgent clinical needs were excluded at presentation, introducing potential selection bias. A major methodological limitation is the mean interval of days between the GP and cardiologist assessments. Pulmonary congestion is a dynamic condition, and during this period, symptoms may evolve, B-lines may partially or fully resolve, and diuretic therapy may be initiated. Consequently, the specialist assessment may represent a retrospective rather than contemporaneous comparator, introducing temporal bias that could attenuate agreement between GP- and cardiologist-derived B-lines measurements and influence estimates of diagnostic performance metrics. Although this effect was explored in an additional stratified analysis, temporal variation remains an inherent constraint of real-world referral pathways and should be considered when interpreting B-line-based diagnostic performance. Although rigorous blinding procedures were applied, incorporation bias cannot be completely excluded. During FCU, GPs relied on visual estimation of left ventricular systolic function, which is less sensitive to subtle abnormalities and cannot diagnose heart failure with preserved ejection fraction. Furthermore, the handheld convex transducer used in this study, while pragmatic for primary care, provides lower spatial resolution than dedicated phased-array probes. The inclusion of multimorbid patients enhances real-world relevance but introduces potential diagnostic confounding, as conditions such as chronic kidney disease may influence B-line formation or complicate interpretation. Finally, the study did not include longitudinal follow-up and therefore cannot evaluate the prognostic implications of these PoCUS examinations. In addition, a formal *a priori* sample size calculation was not performed, as the study was conceived as a pragmatic diagnostic accuracy study, and the sample size was determined by feasibility rather than predefined power assumptions.

### 4.4 Overall conclusion

Our findings suggest that, following brief focused training, GPs may be able to use PoCUS to support the identification of HF in patients presenting with new-onset dyspnea in real-world primary care settings. The use of PoCUS was associated with higher diagnostic accuracy, greater agreement with cardiologist-adjudicated HF status, and more appropriate case reclassification. These findings indicate a potential role for PoCUS as a complementary diagnostic tool in primary care, with implications for clinical decision-making and referral practices.

## Data Availability

The original contributions presented in the study are included in the article/Supplementary material, further inquiries can be directed to the corresponding author.

## Glossary

A4Ch: apical four-chamber view
AUC: area under the curve
ACE: angiotensin-converting enzyme
ALS: amyotrophic lateral sclerosis
ARB: angiotensin II receptor blocker
CE: Conformité Européenne
CI: confidence interval
COPD: chronic obstructive pulmonary disease
DOAC: direct oral anticoagulant
ECG: electrocardiogram
E/e’: early mitral inflow velocity to mitral annular early diastolic velocity ratio
EACVI: European Association of Cardiovascular Imaging
ESC: European Society of Cardiology
FDA: Food and Drug Administration
GP: general practitioner
HF: heart failure
HFpEF: heart failure with preserved ejection fraction
HFrEF: heart failure with reduced ejection fraction
IBM SPSS: International Business Machines Statistical Package for the Social Sciences
ICC: intraclass correlation coefficient
LUS: lung ultrasound
LVEF: left ventricular ejection fraction
N/A: not applicable
NPV: negative predictive value
NS: not significant
NT-proBNP: N-terminal pro-B-type natriuretic peptide
P2Y12: platelet ADP receptor
PoCUS: point-of-care ultrasound
PPV: positive predictive value
ROC: receiver operating characteristic
TAG: thrombocyte aggregation inhibitor
VKA: vitamin K antagonist
