# Supplementary material for: Point-of-care ultrasound improves the diagnosis of heart failure in patients with dyspnea in primary care
Source: Front Med (Lausanne). 2026 Feb 13;13:1721066. doi: 10.3389/fmed.2026.1721066 (PMC12947844; doi:10.3389/fmed.2026.1721066)
Supplement: Supplementary file 1 [file Data_Sheet_1.DOCX]

Supplementary Material

## Supplementary Figures

**Supplementary Figure 1.** Receiver Operating Characteristic (ROC) curves illustrating the diagnostic performance of NT-proBNP and GP-assessed B-line count for heart failure. Both parameters demonstrate excellent discriminatory capacity, as reflected by their high area-under-the-curve (AUC) values, supporting their utility in identifying patients with heart failure. NT-proBNP: N-terminal pro-B-type natriuretic peptide.

**Supplementary Figure 2.** Distribution of four diagnostic parameters in patients with and without a final diagnosis of heart failure: GP-assessed B-line count (A, upper left panel), cardiologist-assessed B-line count (B, upper right panel), NT-proBNP level (C, lower left panel) and early mitral inflow velocity to mitral annular early diastolic velocity ratio (E/e’) (D, lower right panel). All four boxplots show significantly higher values of these parameters in patients with heart failure than in those without (p<0.001 for all), highlighting their diagnostic relevance and supporting the clinical validity of GP decision-making following PoCUS. HF: heart failure.

## Supplementary Tables

| Patient characteristics and clinical variables | All patients (n=102) | Patients with cardiologist-confirmed HF (n=68) | Patients without cardiologist-confirmed HF (n=34) | p value |
| --- | --- | --- | --- | --- |
| Vital parameters |  | | | |
| Heart rate, beats/min | 80.2±13.3 | 79.3±12.4 | 82.2±14.6 | NS |
| Systolic arterial blood pressure, mmHg | 138.6±15.9 | 138.8±16.2 | 138.1±15.3 | NS |
| Diastolic arterial blood pressure, mmHg | 81.2±10.9 | 81.3±10.5 | 81.2±11.7 | NS |
| Conditions |  | | | |
| Dyslipidaemia | 53 (52.0%) | 37 (69.8%) | 16 (30.2%) | NS |
| Laboratory parameters |  | | | |
| Creatinine clearance, ml/min/1.73m^2^ |  | | | |
| <30 | 3 (2.9%) | 3 (100%) | 0 (0%) | NS |
| 30-59 | 30 (29.4%) | 26 (86.7%) | 4 (13.3%) | 0.006 |
| ≥60 | 69 (67.7%) | 39 (56.5%) | 30 (43.5%) | 0.002 |
| Hemoglobin, g/l | 129.2±17.9 | 130.6±16.0 | 126.5±21.0 | NS |
| Regular medications |  | | | |
| ACE inhibitors | 62 (60.8%) | 42 (67.7%) | 20 (32.3%) | NS |
| Angiotensin II receptor blockers | 20 (19.6%) | 15 (75.0%) | 5 (25.0%) | NS |
| Calcium antagonists | 44 (43.1%) | 35 (79.6%) | 9 (20.4%) | 0.02 |
| Alpha_1_ receptor antagonists | 13 (12.7%) | 12 (92.3%) | 1 (7.7%) | NS |
| Imidazolin-I-1 agonists | 6 (5.9%) | 6 (100%) | 0 (0%) | NS |
| Beta-blockers | 68 (66.7%) | 51 (75.0%) | 17 (25.0%) | 0.012 |
| Diuretics (loop; thiazide; thiazide-like diuretics) | 37 (36.3%) | 24 (64.9%) | 13 (35.1%) | NS |
| Aldosteron antagonists | 0 (0%) | 0 (0%) | 0 (0%) | N/A |
| Statins | 50 (49.0%) | 34 (68.0%) | 16 (32.0%) | NS |
| Fibrates | 5 (4.9%) | 4 (80.0%) | 1 (20.0%) | NS |
| TAGs (aspirin; P2Y12 inhibitors) | 37 (36.3%) | 24 (64.9%) | 13 (35.1%) | NS |
| Oral anticoagulants (VKAs; DOACs) | 30 (29.4%) | 29 (96.7%) | 1 (3.3%) | <0.001 |
| Antianginal agents (e.g., nitrates, trimetazidine) | 16 (15.7%) | 14 (87.5%) | 2 (12.5%) | NS |
| Digoxin | 0 (0%) | 0 (0%) | 0 (0%) | N/A |
| Amiodarone | 2 (2.0%) | 2 (200%) | 0 (0%) | NS |

**Supplementary Table 1.** Supplementary data of further patient characteristics in the overall study population, stratified by final diagnosis of heart failure.

Values are present as mean ± standard deviation (SD) or number (%). HF: heart failure; ACE: angiotensin-converting enzyme; ARB: angiotensin II receptor blocker; TAGs: thrombocyte aggregation inhibitors (aspirin, P2Y12 inhibitors); VKAs: vitamin K antagonists; DOACs: direct oral anticoagulants; N/A: not applicable; NS: not significant.

| Clinical and echocardiographic parameters | All patients (n=102) | Patients with cardiologist-confirmed HF (n=68) | Patients without cardiologist-confirmed HF (n=34) | p value |
| --- | --- | --- | --- | --- |
| Modified Borg Dyspnea Scale, points (0-10) | 3.3±2.3 | 3.8±2.4 | 2.3±1.8 | <0.001 |
| Interventricular septal thickness, mm | 11.0±2.0 | 11.5±2.1 | 10.9±1.5 | NS |
| Left ventricular end-diastolic diameter, mm | 51.5±7.4 | 53.0±7.9 | 48.4±5.0 | 0.005 |
| Right ventricular basal diameter, mm | 39.4±7.8 | 41.0±8.0 | 35.2±5.2 | 0.004 |
| Posterior wall thickness, mm | 10.7±1.3 | 10.9±1.4 | 10.3±1.1 | NS |
| Left atrial volume, ml | 91.8±47.3 | 101.0±51.5 | 71.7±27.2 | 0.013 |
| Inferior vena cava diameter, mm | 20.0±6.0 | 20.6±6.1 | 17.4±4.6 | NS |
| Left ventricular ejection fraction, % | 60.3±14.2 | 56.3±15.3 | 68.3±6.5 | <0.001 |
| Estimated pulmonary artery systolic pressure, mmHg | 32.2±15.7 | 36.2±16.6 | 23.2±8.2 | <0.001 |
| E/e’ | 10.7±5.5 | 12.9±6.3 | 7.8±1.7 | <0.001 |
| Total number of B-lines (all GPs) | 11.8±12.0 | 16.1±12.5 | 3.4±3.2 | <0.001 |
| Total number of B-lines (cardiologist) | 10.2±12.6 | 13.7±13.9 | 3.1±4.2 | <0.001 |

**Supplementary Table 2.** Evaluation of heart failure-related clinical and echocardiographic parameters in the overall cohort and by final heart failure diagnosis.

**Values are presented as mean ± standard deviation (SD). HF: heart failure; E/e′: early mitral inflow velocity to mitral annular early diastolic velocity ratio. NS: not significant.**

| Non-cardiac cause | n (%) |
| --- | --- |
| COPD | 8 (23.5%) |
| Asthma bronchiale | 5 (14.7%) |
| Obesity | 4 (11.8%) |
| Anaemia | 4 (11.8%) |
| Psychogenic causes | 4 (11.8%) |
| Pneumonia | 3 (8.8%) |
| Interstitial lung disease | 2 (5.9%) |
| Polyarthritis | 2 (5.9%) |
| Spondylitis ankylopoetica | 1 (2.9%) |
| Rib fracture | 1 (2.9%) |

**Supplementary table 3.** Non-cardiac causes of dyspnea in patients without a final diagnosis of heart failure. The table summarizes the full spectrum of alternative etiologies identified among non-HF patients. These data highlight the clinical heterogeneity of dyspnea presentations in primary care. COPD: chronic obstructive pulmonary disease.
