# Supplementary figures and images for: Point-of-care ultrasound improves the diagnosis of heart failure in patients with dyspnea in primary care
Source: Front Med (Lausanne). 2026 Feb 13;13:1721066. doi: 10.3389/fmed.2026.1721066 (PMC12947844; doi:10.3389/fmed.2026.1721066)

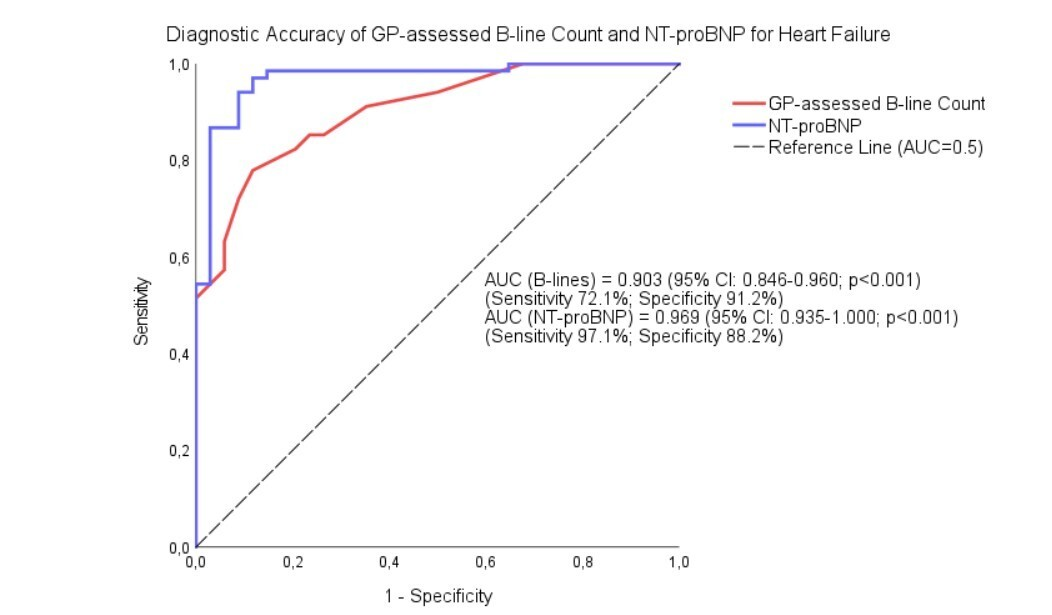

Supplement: Supplementary file 2 [file Image_1.TIF]

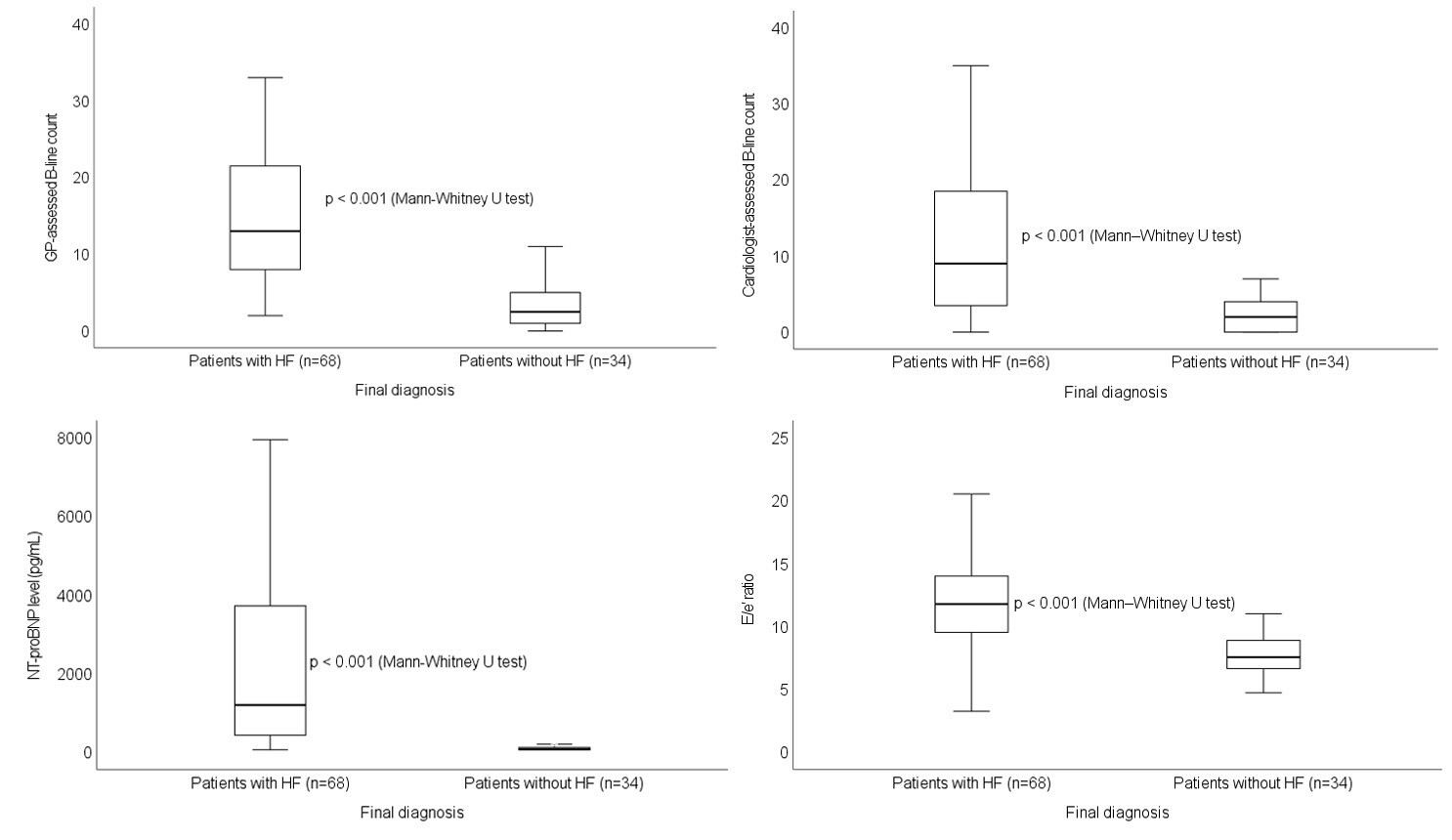

Supplement: Supplementary file 3 [file Image_2.TIF]
